# Flexible Ecoflex^®^/Graphene Nanoplatelet Foams for Highly Sensitive Low-Pressure Sensors

**DOI:** 10.3390/s20164406

**Published:** 2020-08-07

**Authors:** Marco Fortunato, Irene Bellagamba, Alessio Tamburrano, Maria Sabrina Sarto

**Affiliations:** 1 Nanotechnology Research Center Applied to Engineering (CNIS), Sapienza University of Rome, 00185 Rome, Italy; irene.bellagamba@uniroma1.it (I.B.); alessio.tamburrano@uniroma1.it (A.T.); mariasabrina.sarto@uniroma1.it (M.S.S.); 2 Department of Astronautical, Electrical and Energy Engineering (DIAEE), Sapienza University of Rome, 00184 Rome, Italy

**Keywords:** low-pressure sensor, foam, graphene, nanoplatelets, Ecoflex^®^, positive piezoresistivity, negative piezoresistivity, wearable devices

## Abstract

The high demand for multifunctional devices for smart clothing applications, human motion detection, soft robotics, and artificial electronic skins has encouraged researchers to develop new high-performance flexible sensors. In this work, we fabricated and tested new 3D squeezable Ecoflex^®^ open cell foams loaded with different concentrations of graphene nanoplatelets (GNPs) in order to obtain lightweight, soft, and cost-effective piezoresistive sensors with high sensitivity in a low-pressure regime. We analyzed the morphology of the produced materials and characterized both the mechanical and piezoresistive response of samples through quasi-static cyclic compression tests. Results indicated that sensors infiltrated with 1 mg of ethanol/GNP solution with a GNP concentration of 3 mg/mL were more sensitive and stable compared to those infiltrated with the same amount of ethanol/GNP solution but with a lower GNP concentration. The electromechanical response of the sensors showed a negative piezoresistive behavior up to ~10 kPa and an opposite trend for the 10–40 kPa range. The sensors were particularly sensitive at very low deformations, thus obtaining a maximum sensitivity of 0.28 kPa^−1^ for pressures lower than 10 kPa.

## 1. Introduction

In recent years, the growing demand for novel, highly flexible, and sensitive pressure sensors has been mainly driven by the great interest towards soft robotics for human cooperation and rehabilitation as well as wearable electronics for human healthcare and activity monitoring [1,2].

A pressure sensor converts a strain/pressure stimulus into an electrical signal. The transduction mechanism can be based on different physical effects, such as capacitive coupling, piezoresistivity, piezoelectricity, or triboelectricity [1,3]. Capacitive sensors are particularly interesting for applications with low power consumption and can monitor static deformations with high sensitivity. However, they are characterized by a limited detection range of strain. Piezoelectric and triboelectric sensors can operate without any external power supply, but they are able to detect only dynamic deformations. Piezoresistive sensors are able to convert the stimulus into an electrical resistance variation. They can detect both transient and static deformations, are low in cost, can be fabricated through simple manufacturing processes, require simple electronic circuits for easy acquisition, and have fast response and a wide detection range [4].

However, conventional piezoresistive pressure sensors that are based on single-crystal silicon, metals, metal oxide, and nitride materials are not suitable for applications requiring soft, stretchable, and squeezable sensing devices due to their rigid and brittle nature. In contrast, new elastomeric nanocomposites that combine the properties of carbon nanotubes (CNTs) [5,6], Ag nanowires or nanoparticles [7], graphene [8], graphene oxide (GO) [9], or graphene nanoplatelets (GNPs) [10] with those of different polymers, such as polydimethylsiloxane (PDMS) [11,12], Ecoflex^®^ [13,14], rubber [15,16], or polyurethane (PU) [17], have recently shown high flexibility and excellent responsiveness to torsion, tension, and compression.

Unfortunately, these kinds of materials can be barely exploited when extremely lightweight and sensitive devices, able to detect pressures lower than some tens of kPa, are required.

Generally, the value of pressure measured by a sensor is organized into four ranges depending on the specific application: ultralow pressure (lower than 1 Pa), subtle pressure (from 1 Pa to 1 kPa), low pressure (from 1 to 10 kPa), and medium pressure (from 10 to 100 kPa) [1,18]. The particular interest in a low-pressure regime covering intrabody pressures and pressures created by gentle manipulation of items [1] has prompted many researchers to develop new ultraflexible piezoresistive sensors capable of detecting ever-lower external stimuli.

Very recently, graphene-based foams with open cells have been demonstrated to possess outstanding mechanical and piezoresistive properties [19,20,21,22,23,24,25,26,27,28,29,30]. Different methodologies have been developed in order to create sensors based on three-dimensional (3D) freestanding interconnected graphene networks or elastomeric foams internally coated with graphene. Chemical vapor deposition (CVD) [31], self-assembly [32,33], freeze-drying [34], and liquid-phase processing [27] are the most commonly adopted techniques. In particular, GNPs have attracted a lot of interest to produce highly sensitive piezoresistive porous sensors also due to fact that they can be produced at low cost and in high quantities for large-scale applications through the thermochemical exfoliation of intercalated graphite compound. For example, in [20], we showed the possibility of obtaining a new flexible piezoresistive material constituted by a GNP-coated PDMS skeleton, which is of interest for medium-pressure applications.

In this study, we developed, for the first time, novel conducting polymeric Ecoflex^®^ foams loaded with GNPs as piezoresistive low-pressure sensors. In recent literature, to the best of our knowledge, there have only been studies concerning 2D strain sensors using Ecoflex^®^ loaded with carbon nanotubes [13]. The use of porous Ecoflex^®^/graphene 3D composite as a pressure sensor has not been investigated yet. In particular, we decided to use Ecoflex^®^ polymer due to its low Young’s modulus (~90 kPa), high mechanical compliance, and strong interfacial bonding with GNPs. 

We focused our attention firstly on evaluating their electromechanical response by applying a quasi-static compression load up to 40 kPa. This investigation revealed that the piezoresistive behavior of the sensors was characterized by two different regimes: a negative piezoresistive behavior for low pressures up to ~10 kPa and a positive piezoresistive trend for higher pressures. Then, in order to investigate the stability of the foams as low-pressure sensors (P < 10 kPa), we performed quasi-static cyclic load tests up to 10 kPa. We also evaluated the sensitivity of these sensors. The obtained maximum sensitivity of 0.28 kPa^−1^ for pressures lower than 10 kPa is one of the highest values when compared to those reported in the literature. Table 1 shows the maximum sensitivity within specific pressure ranges of different polymeric foams/sponges loaded with graphene-based nanostructures and used as pressure sensors. Furthermore, the production method and the application of the produced devices are included. It can be seen that our sensor was produced with one of the simplest and most cost-effective manufacturing processes.

## 2. Materials and Fabrication Methods

### 2.1. Ecoflex^®^ Foam Production

The first step of the fabrication process, based on the procedure developed in our previous study [20], was the production of Ecoflex^®^ open cell foams, obtained as replica of the inverse structure of leachable templates, made of dark brown sugar particles with an average grain size of 270 µm. The templates were prepared in a metallic mold in which the sugar was compressed for ~1 min at constant pressure of ~309 mbar. The Ecoflex^®^ rubber was prepared by mixing the prepolymer (Smooth-On Part A) and the curing agent (Smooth-On Part B) with a weight ratio of 1:1 for 5 min. In some cases, we also added a silicone thinner fluid with a concentration of 8 wt % of the total amount of the mixture (Part A + Part B) in order to improve the elasticity and reduce the viscosity of the material. Furthermore, to remove the air entrapped during the mixing procedure, the mixture was left to rest inside a desiccator at a pressure of ~313 mbar for 15 min. The cylindrical-shaped templates were extracted from the mold and placed on an aluminum dish. The dish was then completely filled with the polymer mixture to infiltrate the sugar-based templates. The curing process was performed at room temperature (T = 24 °C) for 12 h, maintaining a constant pressure of ~313 mbar. At the end of the curing process, after removing the excess polymer from the surfaces of the Ecoflex^®^/sugar pillars, the sugar porogens were leached out in deionized water inside an ultrasonic bath for 1 h. 

**Figure 1:**
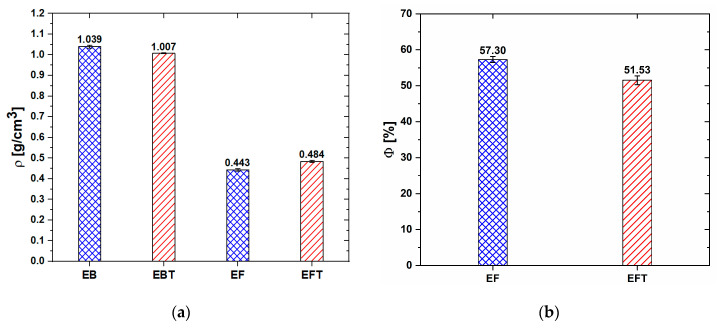
Density (**a**) and porosity (**b**) of the produced bulk and foam samples.

The obtained cylindrical open cell foams had an average diameter of ~11.5 mm and thickness of ~6.5 mm. Their porosity can be evaluated using the following equation [35]:

(1) $$\Phi =1-\rho_{foam}/\rho_{Ecoflex}$$

where $\rho_{Ecoflex}$ is the density of either the Ecoflex^®^ bulk (EB) or the Ecoflex^®^ thinner bulk (EBT), and $\rho_{foam}$ is the apparent density of either the Ecoflex^®^ foam (EF) or the Ecoflex^®^ thinner foam (EFT). The density values are reported in Figure 1a. In particular, the values of $\rho_{Ecoflex}$ were obtained from the measured weight and volume of the bulk samples. The apparent density of both foam types (EF and EFT), evaluated as the mass of the polymer 3D skeleton divided by the total volume including porosity, was around two times lower than the density of bulk samples (EB and EBT). Moreover, as shown in Figure 1b, the porosity of EF samples, calculated with (1), was nearly 10% higher than the one obtained for EFT samples. This can be attributed to the low viscosity of Ecoflex^®^ with thinner causing better polymer infiltration of templates.

### 2.2. Ecoflex^®^ Foam Infiltration Process

In order to obtain electrically conductive porous elastomeric samples, the foams were loaded with GNPs dispersed in ethanol by a drop-casting method. 

The nanofillers were obtained by exfoliating commercial graphite nanoparticles in ethanol at a temperature of 5 °C. In particular, the exfoliated GNPs had an average thickness of 8–10 nm and lateral dimensions up to a few micrometers, as already shown in [20,36,37,38]. After the exfoliation, the colloidal suspension was boiled to promote the partial evaporation of the solvent with the aim of obtaining a specific higher GNPs/ethanol weight ratio. In particular, we used four different concentrations for the foam infiltration: 1, 1.5, 2, and 3 wt %. Lower GNP weight ratios were not considered because of the resistive response of samples with 1 wt % of GNPs being too high (see next paragraph). On the other hand, higher concentrations of GNPs showed issues concerning both material processing and the infiltration step.

**Figure 2:**
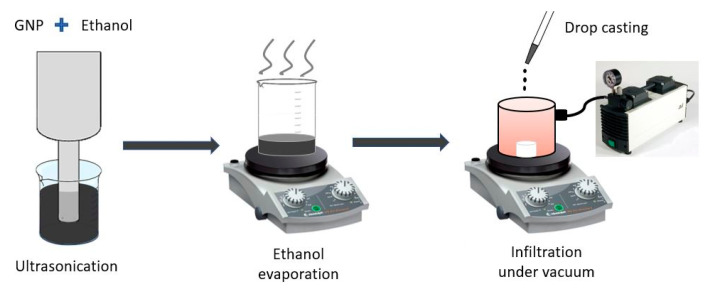
Schematic representation of the foam infiltration process.

During the entire infiltration process, the suspension was stirred in a sonicator bath in order to avoid nanofillers reagglomeration. The suspension was dropped on foams put inside a vacuum cylinder and over a hot plate (maintained at a temperature of 50 °C). In total, 20 drop-casting cycles of 50 µL of suspension were performed. At the end of each drop-casting cycle, the cylinder was sealed with a hermetic cup, and the infiltration process took place for 60 s at a pressure of ~213 mbar. The vacuum facilitated the evaporation of the solvent by lowering its boiling point (from 78 to 50 °C), thus avoiding the possible degradation of the polymer chains. The infiltration process is schematically represented in Figure 2.

**Figure 3:**
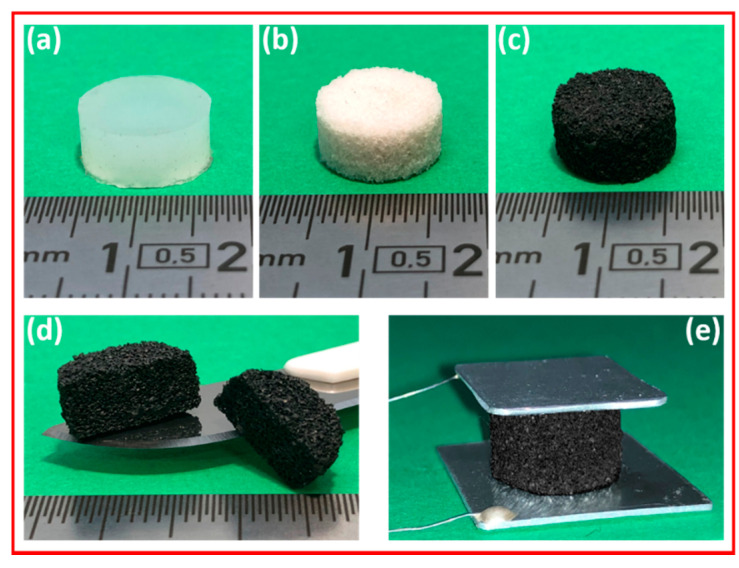
Ecoflex^®^-based samples: bulk sample (**a**), open cell foam (**b**), GNP-loaded foam (**c,d**), and GNP-loaded foam with the conductive plates and wires (**e**).

Finally, the infiltrated foams were sandwiched between two thin aluminum (Al) conductive plates, working as electrodes. The Al plates were glued to the foams using a conductive silver epoxy adhesive. It can be noticed that the precompression due to the presence of the plates was quite low, considering that $P_{plate}=m_{plate}*g/\pi *r^{2}\sim 0.09\;\mathrm{kPa}$, where $P_{plate}$ is the applied pressure due to the use of the Al plates, $m_{plate}$ is the mass of the top plate, *g* is the gravitational acceleration, and r is the foam radius. Then, two wires, used for the electrical tests, were attached, one for each electrode by means of the conductive glue. Figure 3 shows an Ecoflex^®^ bulk sample (Figure 3a), an Ecoflex^®^ foam (Figure 3b), a foam infiltrated with GNPs (Figure 3c,d), and the sponge with the conductive plates and wires (Figure 3e), ready for electromechanical characterizations.

## 3. Characterization and Testing

### 3.1. Morphological Characterization

Field-emission scanning electron microscopy (FE-SEM, Auriga, Carl Zeiss, Oberkochen, Germany) operating with an accelerating voltage of 2 kV was used to investigate the foam morphology before and after GNP infiltration. Prior to SEM imaging, noninfiltrated foams were metalized with 20 nm of Cr using a Quorum Technologies Q150T ES sputter coater (Laughton, East Sussex, UK) in order to prevent surface charging. 

**Figure 4:**
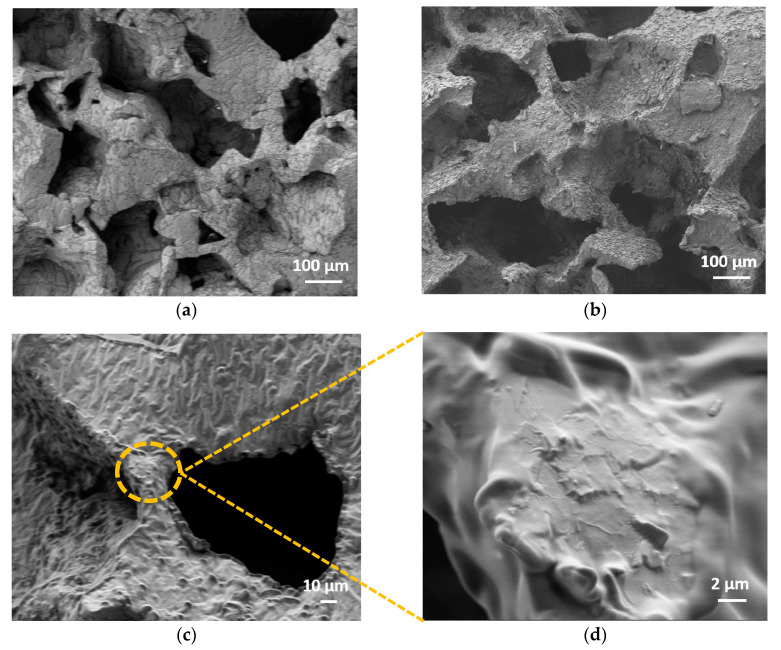
Cross section of an Ecoflex^®^ foam (EF) sample (**a**) and an EF-3% (**b**). Magnification of the EF-3% foam (**c**) and the detail of some embedded and partially emerging GNPs (**d**).

Figure 4a shows the cross section of an Ecoflex^®^ foam as obtained after leaching. The polymeric structure displayed the negative replica of the sugar particles. The pores had dimensions of a few hundreds of micrometers and were constituted by microperforated walls, making the produced foams open-cell structures. Figure 4b–d shows the cross section of an infiltrated Ecoflex^®^ foam. GNPs over the surface of the foam pores formed an electrically conductive network responsible for the piezoresistive response of the material. Besides, as highlighted in Figure 4c,d, GNPs were partially incorporated in the elastomer. The filler integration took place during the infiltration step. Although Ecoflex^®^ is highly compatible with ethanol [39], during drop-casting, the presence of the solvent induced blistering and solvation of the polymer, which made it softer and sticker, thus promoting the partial embedding of the GNPs. The obtained 3D structure improved the sensor response stability compared to the pressure sensors previously reported in [20].

### 3.2. Mechanical Characterizations

The different Ecoflex^®^ samples were mechanically characterized by applying a quasi-static cyclic compressive load at room temperature and constant humidity using a universal testing machine (Instron 3366, ITW Test and Measurement Italia S.r.l., Instron CEAST Division, Pianezza, Italy), equipped with a 10 N load cell [20]. The barreling effect [40] due to friction on the Ecoflex^®^ bulk samples was minimized by lubricating the parallel surfaces of the polymeric cylinders. Moreover, the presence of a toe region at the beginning of the stress–strain curve was properly compensated after tests.

**Figure 5:**
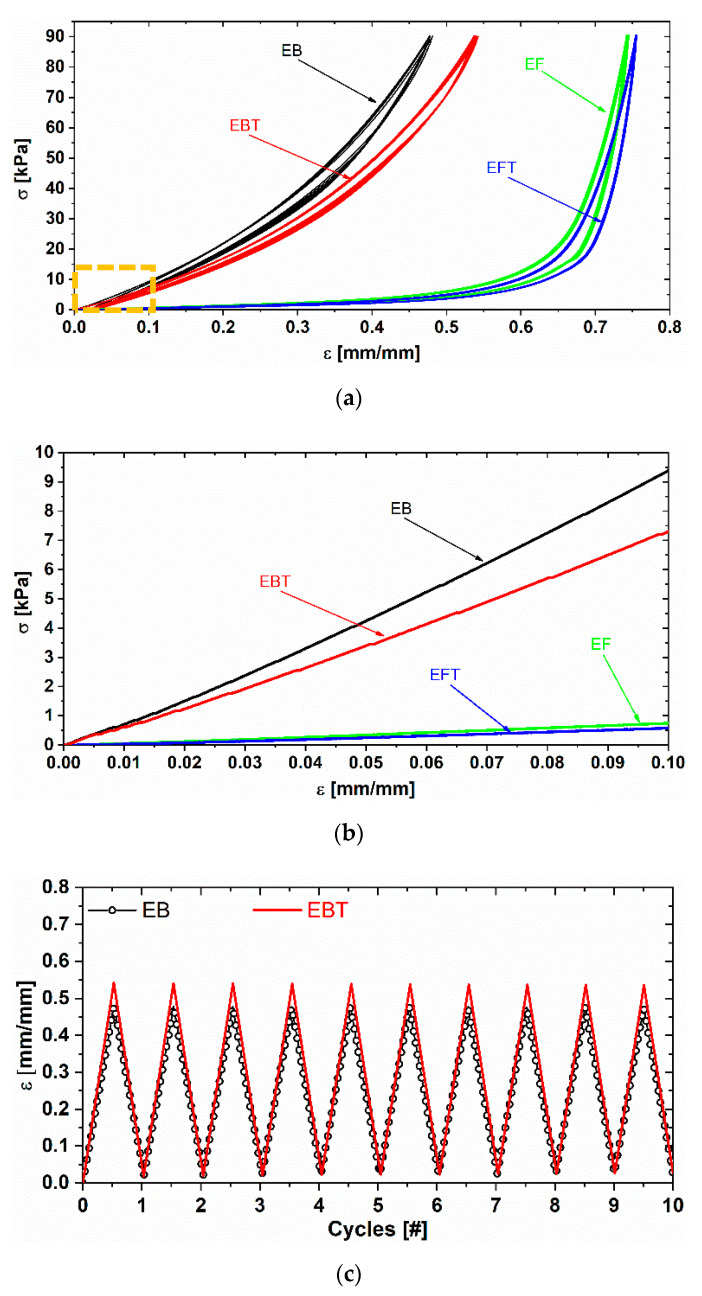

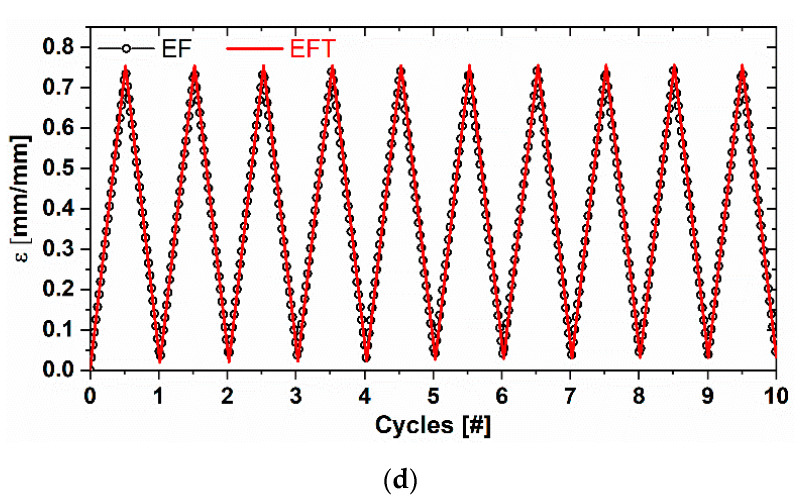
Stress–strain curves (**a**), magnification of the linear region of the stress–strain curves (**b**), and deformation as a function of the cyclic compressive loads (**c,d**) for different Ecoflex^®^ samples during cyclic compression tests.

All the bulk and foam samples (EB, EBT, EF, and EFT) were subjected to uniaxial quasi-static compression tests, each of which constituted 10 loading/unloading cycles, performed at a crosshead speed of 1 mm/min. The maximum stress of each cycle was set at 90 kPa. The loading/unloading stress–strain curves of the tested samples are reported in Figure 5a,b; Figure 5c,d shows the strain variation during the 10 loading/unloading cycles.

The stress–strain curves reported in Figure 5a clearly highlight the higher compressibility in the entire investigated deformation range of the EBT and EFT samples with respect to the EB and EF ones, respectively, thanks to the use of the thinner fluid, which lowered the stiffness of the cured Ecoflex^®^ rubber. As a consequence, EBT and EFT samples reached higher deformations at the same maximum stress level. The existence of small hysteresis loops (area between loading and unloading curves) are related to the material viscoelastic properties [41]. Figure 5c,d proves that all the produced samples were characterized by a steady mechanical behavior.

**Figure 6:**
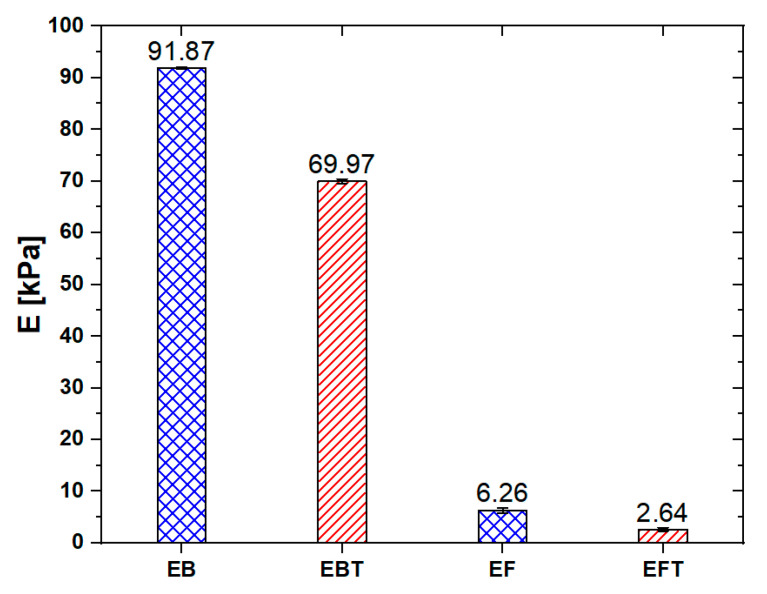
Compressive Young’s modulus of the Ecoflex^®^ bulk (EB) and EF samples.

In order to better characterize the influence of thinner fluid on the elastic material properties, the compressive Young’s modulus (E) of each sample was evaluated from the initial slope of the stress–strain curve at 0.1% deformation within the linear region highlighted in Figure 5b. As shown in the column chart in Figure 6, the E of the EB samples was reduced by a factor of ~3 and by a factor of ~1.3 for EF samples when 8 wt % of thinner fluid was added to the polymer.

**Figure 7:**
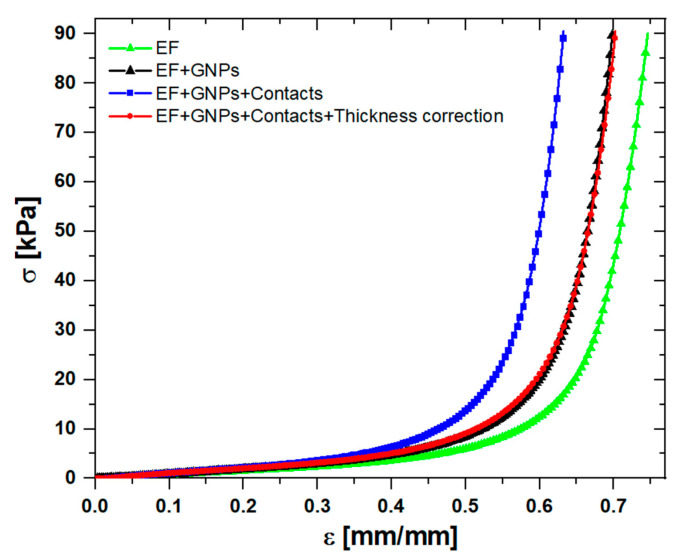
Differences in the mechanical behavior of the foam with and without the electrical contacts.

It should be noted that the mechanical behavior of foams can be affected by the infiltration of GNPs. This occurs because the Ecoflex^®^ foam is highly flexible and even a little amount of GNP partially embedded in the polymer can increase the stiffness of the structure. Indeed, as reported in Figure 7, the foam loaded with GNPs (black line) appeared more rigid than the EF (green line). We also observed that the application of Al electrodes to the loaded foam further affected the mechanical response. The foam with electrodes (blue line) was considerably more rigid than the loaded foam without electrodes (black line). This can be explained by considering the penetration inside the loaded foam of the conductive epoxy adhesive, which was used to attach the Al plates. By analyzing the micrograph images of the foams bonded to the Al electrodes, we found that the penetration of the conductive epoxy adhesive was ~0.5 mm on each side of the sample. By assuming that the mixed foam-conducting adhesive region was substantially more rigid than the EF and by subtracting the value of ~1 mm from the initial value of the loaded foams’ thickness, we obtained the red stress–strain curve, which perfectly overlapped the curve relative to the loaded foam without electrodes (see Figure 7). 

### 3.3. Electrical Characterizations

The electrical resistance (R_0_) of the foams after GNP infiltration was measured using a two-wire volt-amperometric technique through a Keithley 6221 DC/AC current source (Keithley, Cleveland, OH, USA) and a Keithley 2182 nanovoltmeter (Keithley, Cleveland, OH, USA) at rest condition, that is, when no mechanical solicitation was applied to the material. The values of R_0_ of foams filled with different weight fraction of GNPs (i.e., 1, 1.5, 2, and 3 wt %), as described in Section 2.2, are reported in Table 2.

It can be observed that when the GNP weight fraction increased, the resistance of the foams decreased. Moreover, it can be seen that for the same amount of nanofiller, the EFT always had a higher value of R_0_ than the one of EF, and that gap increased with increasing GNP concentration. In fact, the use of thinner made the polymer partially solvated by the solvent. Hence, during the infiltration process, ethanol increased the Ecoflex^®^ softening, causing a deeper incorporation of the conductive platelets. The final result was a reduction in the number of contacts between adjacent GNPs and, consequently, the effective electrical conductivity of the foam.

In particular, EFT samples containing lower concentrations of GNPs (i.e., 1 and 1.5 wt %) showed resistance values (of several hundreds of kiloohms) about one order higher compared to those (of same tens of kiloohms) of EF samples with the same filler weight fraction. Because of these very high resistance values, we decided not to consider the samples at 1 and 1.5 wt % of GNPs for other analyses. Besides, in the following section dealing with electromechanical tests and the evaluation of the piezoresistive response of foams, we focused our attention only to Ecoflex^®^ foams with 3 wt % of GNPs (EF-3% and EFT-3%) because the values of the effective electrical conductivity (i.e., 3.7 and 1.6 mS/m for EF-3% and EFT-3%, respectively) were very close to the ones of PDMS-based piezoresistive sensors analyzed in [20].

### 3.4. Electromechanical Characterizations

With the aim of demonstrating the feasibility of exploiting coated Ecoflex^®^ foams with 3 wt % of GNPs as low-pressure sensors (i.e., when P < 10 kPa), we performed several electromechanical tests by subjecting the samples under various quasi-static loading conditions, including cyclic compressions. 

**Figure 8:**
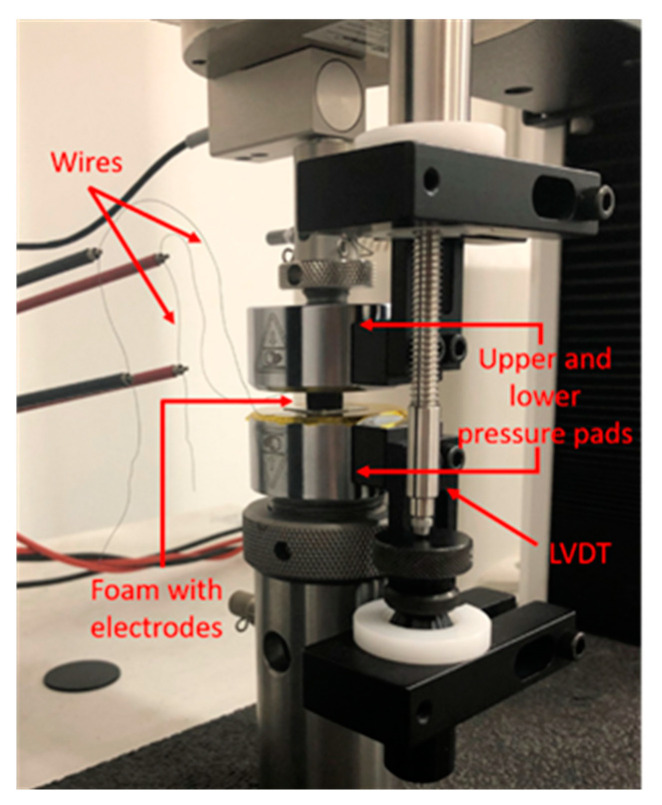
Image of the electromechanical setup.

The piezoresistive response of the sensors was assessed by conducting quasi-static uniaxial compression tests in conjunction with resistance measurements at constant temperature (~21 °C) and constant relative humidity (~43% RH) using the previously described instrumentation. In particular, we injected a DC current with an amplitude of 1 µA and, by measuring the voltage, we evaluated the change of the resistance as a function of the applied load during the execution of mechanical tests. A picture of the test setup is shown in Figure 8.

Firstly, monotonic compression tests with an applied increasing pressure from 0 to 40 kPa and a crosshead speed of 0.1 mm/min were carried out. In particular, three tests were performed in sequence to also investigate the repeatability of the foams’ electrical response. Notably, after the end of each test and load release, the successive test was started only once the resistance reached 99% of its initial value R_0_. In fact, due to viscoelasticity, it was necessary to wait about 10 s between two consecutive tests. 

**Figure 9:**
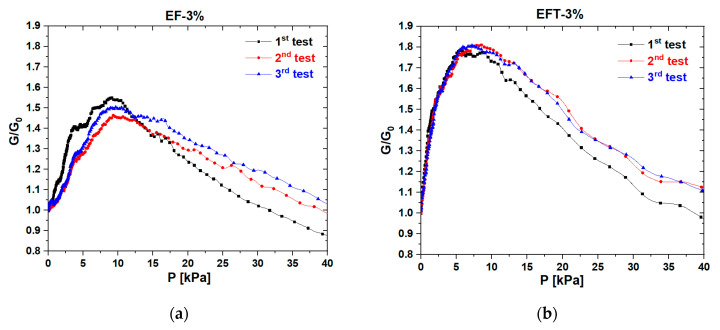
Normalized conductance vs. pressure obtained during three consecutive quasi-static monotonic loading tests at a speed of 0.1 mm/min: response of EF-3% (**a**) and EFT-3% (**b**) samples.

The graphs in Figure 9 show the variation in the normalized conductance G/G_0_ of EF-3% (Figure 9a) and EFT-3% (Figure 9b), G_0_ being the reciprocal of the resistance R_0_ of Table 2. We observed that the piezoresistive response of the EFT-3% was highly repeatable at pressures lower than 10 kPa. The trends shown in Figure 9 also make evident another important aspect: the electromechanical response of the fabricated porous sensors was characterized by two opposite behaviors depending on the pressure range [42,43]. At low pressures, up to ~10 kPa, conductive foams showed a negative piezoresistive effect, that is, their conductance increased with the applied pressure [20,24]. On the contrary, in the range within 10 and 40 kPa, the conductance of the sensors decreased when the compression load increased, demonstrating a positive piezoresistive behavior [44,45]. The inversion of the piezoresistive trend (from negative to positive) occurred for a strain of ~45%.

**Figure 10:**
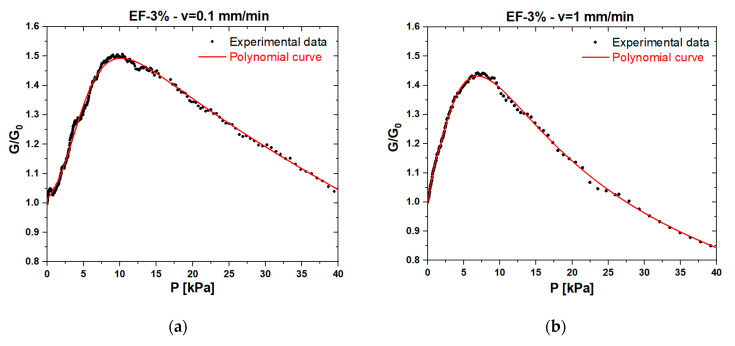
Normalized conductance vs. pressure and corresponding polynomial fitting curves obtained at a speed of 0.1 (**a**) and 1 (**b**) mm/min for the EF-3% sample.

**Figure 11:**
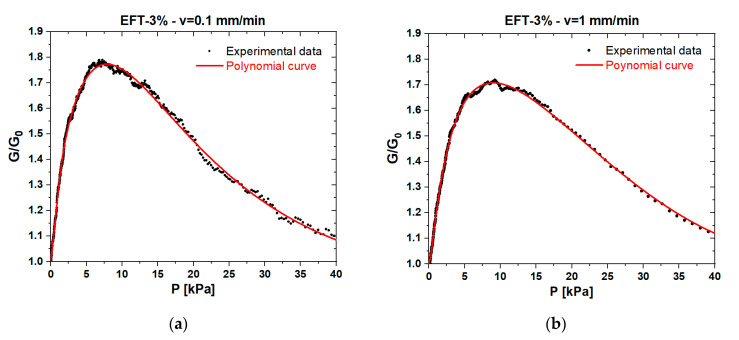
Normalized conductance vs. pressure and corresponding polynomial fitting curves obtained at a speed of 0.1 (**a**) and 1 (**b**) mm/min for the EFT-3% sample.

In order to figure out the influence of applied load speed in a quasi-static regime on the piezoresistive response of foams, we performed electromechanical tests at two different velocities: 0.1 and 1 mm/min. The results are reported in Figure 10 and Figure 11, which show that, in the low-pressure range (P < 10 kPa), the electromechanical response of both EF-3% and EFT-3% was poorly influenced by the speed of the external load. In particular, at lower crosshead speeds, the maximum conductance increased by ~3%.

**Figure 12:**
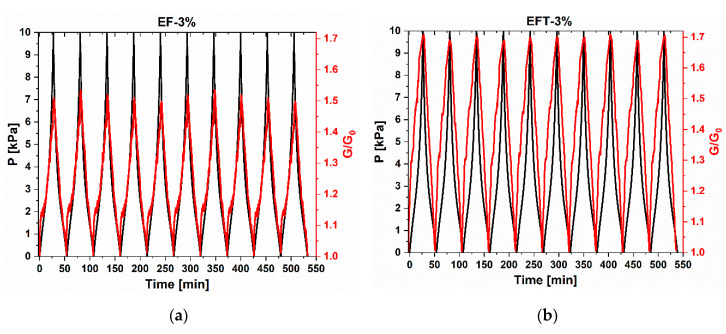
Normalized conductance measured during 10 consecutive loading/unloading tests of the EF (**a**) and EFT (**b**) pressure sensors.

The stability of the sensors within the low-pressure range was examined by performing cyclic loading/unloading compression tests with an applied pressure ranging from 0 to 10 kPa. The results reported in Figure 12 show that both EF-3% and EFT-3% sensors provided repeatable response throughout the cycles. 

To further characterize the performance of the produced Ecoflex^®^/GNP foams, the curves in Figure 10 and Figure 11 were used to evaluate the pressure sensor sensitivity *S*. The experimental G–P curves were firstly fitted with the polynomial functions represented by the red solid lines in the same figures. Then, according to expression (2), the sensitivity (normalized with respect to the initial conductance) was evaluated by calculating the absolute value of the derivative of the fitting polynomials [46]:

(2) $$S=\left|\frac{dG}{dP}\right|\cdot \frac{1}{G_{0}}$$

where *P* is the applied pressure in kPa.

**Figure 13:**
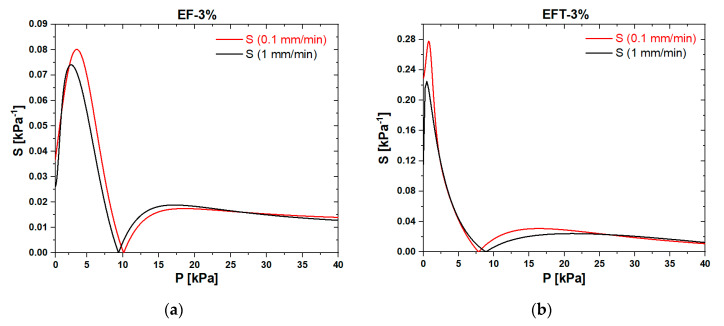
Sensitivity of samples EF-3% (**a**) and the EFT-3% (**b**) at two different crosshead speeds.

The sensitivity of the two types of foams (EF-3% and EFT-3%) as a function of the applied pressure are reported in Figure 13a,b. It can be seen that the sensitivity rapidly increased, reaching, for a speed of 0.1 mm/min, the maximum value of 0.08 kPa^−1^ with the EF-3% sample and a surprising maximum value of 0.28 kPa^−1^ with the EFT-3% one at the pressure of 3.6 and 0.75 kPa, respectively. The maximum value of *S* obtained for 1 mm/min was a little lower than the aforementioned ones. Then, at higher pressures, the sensitivity started to decrease, reaching the zero value in correspondence with the inversion points of piezoresistivity, namely, when P ≅ 10 kPa for the EF-3% sample and when P ≅ 8 kPa for the EFT-3% one. 

Notably, the slightly lower pressure value of the EFT-3% sample can be ascribed to its higher compressibility (i.e., lower Young’s modulus) compared to the one of the foam produced without thinner. Beyond 10 kPa, the sensors’ sensitivity firstly rose until the applied pressure value was nearly 15 kPa. Then, *S* was characterized by a slowly decreasing trend between ~15 and 40 kPa.

## 4. Results and Discussion

The compression tests reported in Section 3.2 have highlighted the high mechanical stability of the proposed elastomeric foams as well as their exceptional flexibility. In fact, the values of Young’s modulus, especially of samples produced with the addition of a small amount of thinner fluid, are extremely low compared with those of other materials reported in the literature [7,21,22,32]. The obtained results make the Ecoflex^®^-based foams extremely promising porous structures for light, flexible, biocompatible, and wearable pressure sensors. This belief led us to investigate the possibility of enhancing the performance of the GNP/PDMS foams developed in [20] in the low-pressure range using the new elastomer. Accordingly, the production and infiltration method was modified and optimized considering the characteristics of Ecoflex^®^. In particular, we selected ethanol as a solvent for the infiltration process due to its great compatibility with elastomer [39]. In contrast to other tested solvents that are too aggressive, ethanol contained in the GNPs-based colloidal suspension seems to be the cause of the slight blistering and solvation of Ecoflex^®^ pores’ surfaces at the used temperature, promoting the partial embedding of the GNPs inside the matrix. In addition, the peculiar shape of GNPs, characterized by sharp edges and large specific surface area, further improve the adhesion between the nanostructures and the Ecoflex^®^ foam [47,48]. Therefore, it is believed that these morphological features have a positive impact on the repeatability of sensors’ output and are responsible for the unique electromechanical behavior of the produced foams. The electrical conductance increased up to ~10 kPa (negative piezoresistivity) and then showed a decreasing trend (positive piezoresistivity) up to the maximum considered pressure value. The experimental characterization demonstrated that higher sensitivity values were obtained in the low-pressure range, where the sensor showed a negative piezoresistive behavior. In particular, the EFT-3% sample showed a maximum sensitivity of 0.28 kPa^−1^ at 3.6 kPa, representing one of the highest values compared to those reported in the literature for foam-based pressure sensors (see Table 1). 

It can be noticed that the performance of the foam produced using thinner fluid was higher not only in terms of flexibility and sensitivity in the low-pressure range but also in repeatable electrical response, as clearly highlighted in Figure 9, showing the variation of the normalized conductance as a function of the applied load during consecutive monotonic compression tests. 

It is commonly known that the mechanism behind the piezoresistive response of either nanocomposites or thin nanostructured films is mainly ascribable to the rearrangement of the conducting filler network when the material is subjected to deformation [20,24,42,43,44,45]. Based on these considerations, we believe that the mechanism responsible for the two different piezoresistivity zones (negative and positive) is related to the morphological characteristics of the produced nanocomposite and the interactions between the nanofiller and the polymer. Indeed, based on the results reported in the literature, a negative piezoresistive behavior is observed when conductive fillers adhere to the cell walls of the porous material [20,24]. On the other hand, as shown in [44,45], a positive piezoresistive response is seen when nanostructures are completely incorporated in the polymer. The partial integration of GNPs in the elastomeric matrix of EF-3% and EFT-3% is assumed to be the cause of the observed particular piezoresistive phenomenon.

**Figure 14:**
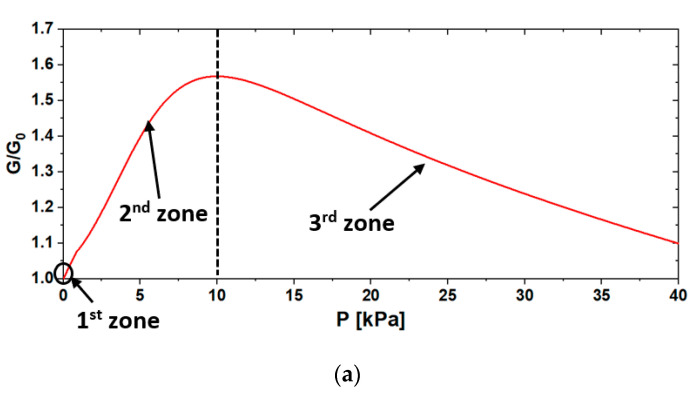

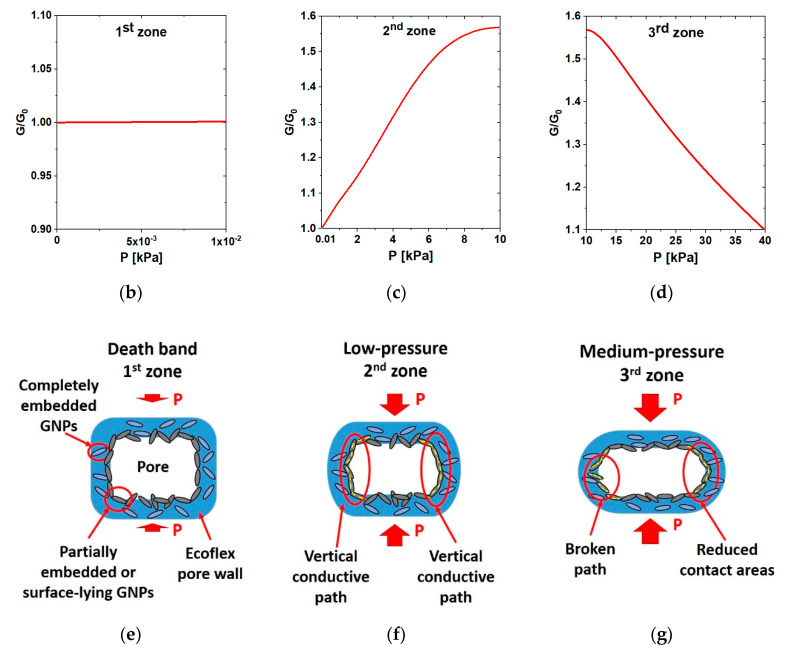
GNP coating modification during compression and its effect on the overall normalized conductance G/G_0_ of the foam. (**a**) Normalized conductance vs. pressure for the EFT-3% sample. (**b**–**d**) Zoom of the death-band zone (1^st^ zone) and the low- (2^nd^ zone) and medium- (3^rd^ zone) pressure ranges of the sensor, respectively. (**e**–**g**) Sketch of the effects of different pressure values on the EFT-3% pore and on the induced modification of GNP percolation paths. In particular, in the sketch, the light blue GNPs represent the completely embedded flakes, while the grey ones represent the partially embedded or surface-lying GNPs.

This aspect is further analyzed in Figure 14 in which, as an example, the output of EFT-3% (Figure 14a) is divided into three zoomed pressure zones (Figure 14b–d). The corresponding Figure 14e–g sketches the effects of the different pressure values on a foam’s pore and on the induced modification of GNP percolation paths. Firstly, the death-band zone of the sensor, in which G/G_0_ remained practically constant up to 0.01 kPa, is represented (Figure 14b). Notice that a similar value was also obtained for EF-3%. Figure 14c,d shows the low- and medium-pressure ranges of the sensor response. In the low-pressure (negative piezoresistance) region, the compression of the Ecoflex^®^ porous structure (Figure 14f) caused a variation in the distance between neighboring GNPs. The conducting sheets came into contact, generating new vertical parallel conducting paths, thus increasing the conductance of the sensor, with the maximum value occurring at ~10 kPa. Under higher pressures (negative piezoresistance) the cylindrical-shaped sample tended to spread in the transversal direction, increasing its cross-sectional area. Thus, the stretched pore walls led to the reorientation of adjacent GNP flakes, breaking some conducting paths, and to an increase in the average electron tunneling distances (Figure 14g) [44,45]. Consequently, the overall conductance of the sensors started to decrease.

## 5. Conclusions

In this work, we developed highly sensitive low-pressure sensors made of Ecoflex^®^ open-cell foams loaded with different concentrations of GNPs. The fast and cost-effective production process started with the preparation of the 3D porous structures. In our case, two sets of polymeric foams were produced. The first one was obtained by preparing a mixture of prepolymer and a curing agent (Part A + Part B), while the second one was prepared by adding 8 wt % of thinner fluid to the mixture in order to improve elasticity and flexibility of the material. The thinner fluid inside the polymer reduced the Young’s Modulus of the final foam by a factor of ~3 with respect to the one without the thinner, increasing its compressibility and maximum deformation. Finally, the cylindrical polymeric foams were infiltrated via the drop-casting method under vacuum using a colloidal suspension of GNPs in ethanol with different concentrations.

In particular, we focused our attention on foams infiltrated with 1 mL of GNPs/ethanol suspension with a concentration of 3 wt % of GNPs because the values of effective electrical conductivity (i.e., 3.7 and 1.6 mS/m for EF-3%, EFT-3%, respectively) were very close to ones of PDMS-based piezoresistive sensors analyzed in our previous work [20].

To characterize the performance of the EF-3% and EFT-3% foams as low-pressure sensors, we performed uniaxial quasi-static electromechanical compression tests, evaluating the variation of the conductance as a function of the applied pressure up to 40 kPa. We observed that the electromechanical behavior of the produced sensors was characterized by two different regimes. Up to ~10 kPa, the conductance increased by increasing the pressure (negative piezoresistive behavior) because the GNPs came into contact, generating new vertical parallel conducting paths. In the range of 10 to 40 kPa, the conductance decreased with increasing compression, showing a positive piezoresistive behavior. The decrease in the conductance was due to the spread in the transversal direction of the cylindrical-shaped sample during compression, which led to the reorientation of adjacent GNP flakes, breaking some conducting paths, and to an increase in the average electron tunneling distances. We speculate that this behavior is possible thanks to the partial embedding of GNPs within the polymer matrix, as the SEM analysis confirmed.

We demonstrated that the EFT-3% and the EF-3% samples had a surprising maximum sensitivity of 0.28 kPa^−1^ at pressure of 0.75 kPa and of 0.08 kPa^−1^ at 3.6 kPa, respectively. Therefore, the proposed graphene/Ecoflex^®^ foams are particularly suitable to be exploited for extremely sensitive low-pressure sensors. 

## Figures and Tables

**Table 1:** Comparison between our sensor and the main pressure sensors present in the literature.

Material	Maximum Sensitivity	Detection Range	Preparation Method	Application (Pressure Range)	Ref.
graphene/Ecoflex^®^ foam	0.28 kPa^−1^	<2.5 kPa	drop-casting	low-pressure sensor	This work
graphene/PDMS foam	0.23 kPa^−1^	50–70 kPa	drop-casting	medium-pressure sensor	[20]
rGO/PI foam	0.18 kPa^−1^ 0.023 kPa^−1^	<1.5 kPa 3.5–6.5 kPa	freeze casting and thermal annealing	low-pressure sensor	[21]
graphene/PU foam	0.26 kPa^−1^ 0.03 kPa^−1^	<2 kPa 2–10 kPa	dip-coating and hydrothermal reduction	low-pressure sensor	[27]
rGO/PI/HT 3D resistive pressure foam	0.36 kPa^−1^	<2 kPa	dip-coating, chemical and thermal reduction	low-pressure sensor	[28]
graphene/PU sponge	1.04 kPa^−1^ 0.12 kPa^−1^	<1 kPa 1–20 kPa	dip-coating and drying	low/medium- pressure sensor	[29]
MWNT/rGO/PU foam	0.022 kPa^−1^ 0.088 kPa^−1^ 0.034 kPa^−1^	<2.7 kPa 2.7–10 kPa >10 kPa	dip-coating	low/medium- pressure sensor	[30]

**Table 2:** Resistance values at rest condition of the foams loaded with different concentrations of graphene nanoplatelets (GNPs).

Foams without Thinner	R_0_ [kΩ]	Foams with Thinner	R_0_ [kΩ]
EF-3%	17	EFT-3%	40
EF-2%	20	EFT-2%	90
EF-1.5%	25	EFT-1.5%	380
EF-1%	30	EFT-1%	510

## Abbreviations

The following abbreviations are used in this manuscript:

GNPs	graphene nanoplatelets
CNTs	carbon nanotubes
GO	graphene oxide
PDMS	polydimethylsiloxane
PU	polyurethane
3D	three-dimensional
CVD	chemical vapor deposition
rGO	reduced graphene oxide
PI	polyimide
HT	heat treatment
MWNT	multiwall carbon nanotubes
EF	Ecoflex® foam without thinner
EFT	Ecoflex® foam with thinner
EB	Ecoflex® bulk without thinner
EBT	Ecoflex® bulk with thinner
FE-SEM	field-emission scanning electron microscopy
DC	direct current
AC	alternating current
Al	Aluminum
